# Assessment of Myocardial Work in Cancer Therapy-Related Cardiac Dysfunction and Analysis of CTRCD Prediction by Echocardiography

**DOI:** 10.3389/fphar.2021.770580

**Published:** 2021-11-11

**Authors:** Jingyuan Guan, Wuyun Bao, Yao Xu, Wei Yang, Mengmeng Li, Mingjun Xu, Yu Zhang, Mei Zhang

**Affiliations:** The Key Laboratory of Cardiovascular Remodeling and Function Research, Chinese Ministry of Education, Chinese National Health Commission and Chinese Academy of Medical Sciences, The State and Shandong Province Joint Key Laboratory of Translational Cardiovascular Medicine, Qilu Hospital of Shandong University, Jinan, China

**Keywords:** anthracycline, trastuzumab, cardiotoxicity, speckle tracking technology, myocardial work

## Abstract

No study has examined myocardial work in subjects with cancer therapy-related cardiac dysfunction (CTRCD). Myocardial work, as a new ultrasonic indicator, reflects the metabolism and oxygen consumption of the left ventricle. The aim of this study was to test the relative value of new indices of myocardial work and global longitudinal strain (GLS) in detecting changes in myocardial function during the treatment of breast cancer by two-dimensional and three-dimensional echocardiography. We enrolled 79 breast cancer patients undergoing different tumor treatment regimens. Follow-up observation was conducted before and after chemotherapy. The effects of breast cancer chemotherapy and targeted therapy on the development of CTRCD [defined as an absolute reduction in left ventricular ejection fraction (LVEF) of >5% to <53%] were detected by two-dimensional and three-dimensional speckle tracking echocardiography. Our findings further indicate that LVEF, myocardial work index (GWI) and myocardial work efficiency (GWE) showed significant changes after the T6 cycle, and GLS showed significant changes after the T4 cycle (*p* < 0.05). The three-dimensional strain changes after T6 and T8 had no advantages compared with GLS. Body mass index (BMI), the GLS change rate after the second cycle of chemotherapy (G2v) and the 3D-GCS change rate after the second cycle of chemotherapy (C2v) were independent factors that could predict the occurrence of CTRCD during follow-up, among which BMI was the best predictor (area under the curve, 0.922). In conclusion, the current study determined that GLS was superior to GWI in predicting cardiac function in patients with tumors with little variation in blood pressure. BMI, G2v and C2v can be used to predict the occurrence of CTRCD.

## Introduction

Cardiovascular disease complicated by chemotherapy has become one of the important causes of posttreatment death in tumor patients (Giordano et al., 2016), among which the most devastating is the heart dysfunction associated with cancer treatment (CTRCD). In the course of treatment, regular follow-up examination is one of the most important methods to prevent cardio-related adverse reactions. Among them, echocardiography has become the most widely used examination method beyond multigated acquisition scanning (MUGA) and magnetic resonance imaging of the myocardium (CMR) due to its advantages including noninvasiveness and repeatability. At present, the most commonly used ultrasonic parameters are left ventricular ejection fraction (LVEF) and global longitudinal strain (GLS). Compared with 2D Speckle Tracking Echocardiogram (2D-STE), the three-dimensional strain measurement has no space limitation and has a more accurate and objective reference value (Zeng et al., 2021).

The left ventricular myocardial work index (GWI) is the area of the left ventricular pressure-strain ring (LV-PSL), which represents the work done by the myocardium from mitral valve closure to mitral valve opening and reflects the metabolism and oxygen consumption of the left ventricle. Its advantage lies in the correction of afterload, which overcomes the dependence of the left ventricular ejection fraction and left ventricular strain on the load (Russell et al., 2012; Hubert et al., 2018). At present, GWI and myocardial work efficiency (GWE) have been confirmed to change earlier than strain in left ventricular remodeling diseases caused by coronary heart disease and structural heart disease (Chan et al., 2019), and the role of myocardial work in the field of oncologic cardiology has not been reported. We followed up breast cancer patients treated with different regimens to explore the value of two-dimensional and three-dimensional speckle tracking in the early monitoring of cardiac function changes and to explore the risk factors for cardiotoxicity caused by breast cancer drug therapy.

## Methods

### Study Population

A total of 79 female patients admitted to Qilu Hospital of Shandong University were enrolled in this study and were divided into three groups according to their treatment plans. Patients in G1 group who received anthracycline-free and non-targeted regimens [Docetaxel (T) 75 mg/m2 on day 1 and Carboplatin (Cb) AUC 6 on day 1, 21 days for a cycle, a total of six cycles]; G2 included patients treated with anthracycline-containing regimens without targeted therapy [Doxorubicin (A) 60 mg/m2 on day 1 or Epirubicin (E) 60–100 mg/m2 on day 1, Cyclophosphamide (C) 600 mg/m2 on day 1, 21 days for a cycle, a total of four cycles; Docetaxel (T) 100 mg/m2 iv on day 1, 21 days as a cycle, a total of four cycles]; G3 included patients treated with anthracyclines combined with targeted drugs [Doxorubicin (A) 50–60 mg/m2 on day 1 or Epirubicin (E) 80–100 mg/m2 on day 1, Cyclophosphamide (C) 600 mg/m2 on day 1, 21 days for a cycle, a total of four cycles; followed by Docetaxel (T) 100 mg/m2 on the first day, 21 days as a cycle, a total of four cycles. At the same time, Trastuzumab (H) was given at the first dose of 8 mg/kg, followed by 6 mg/kg for a period of 21 days, for a total of four cycles. Combined with or without Pertuzumab, first dose of 840 mg and then 420 mg for each period of 21 days, for a period of four cycles]. All patients underwent echocardiography before chemotherapy (T0) and after the end of chemotherapy cycle 2 (T2), cycle 4 (T4), cycle 6 (T6), and cycle 8 (T8).

The patients who met the following inclusion criteria were enrolled: 1) those aged less than 70 years old; 2) patients diagnosed with breast cancer by histology or cytopathology; 3) patients set to receive adjuvant chemotherapy combined with trastuzumab combined with or without pertuzumab; 4) those with no previous history of chemotherapy or radiotherapy. The major exclusion criteria included poor echocardiography image quality that did not allow complete analysis of the ultrasonic data; patients with heart failure, history of coronary heart disease, acute coronary syndrome or myocarditis, myocardial infarction, severe valvular disease, cardiomyopathy and other serious heart disease; and patients with persistent atrial fibrillation and severe arrhythmia. The study protocol was approved by the Ethics Committee of Shandong University Qilu Hospital (Ethics approval number: 2018163), and written informed consent was obtained from all participants.

### Demographic Characteristics

Data on age, body mass index, current or previous smoking, diabetes mellitus, coronary artery disease, systolic blood pressure (SBP), diastolic blood pressure (DBP), and administration of antihypertensive drugs and statins were collected at enrollment. Serum levels of fasting blood glucose (GLU), serum triglycerides, total cholesterol, low-density lipoprotein cholesterol (LDL-C) and high-density lipoprotein cholesterol (HDL-C) were measured in the clinical laboratory departments of Qilu hospitals.

### Echocardiography

All subjects were examined by a GE Vivid E95 or GE Vivid E9 color Doppler ultrasound with an M5S-D probe (probe frequency 1.4–4.6 Hz) and a 4V-D probe (probe frequency 1.5–4.0 Hz). Ultrasound images were collected, and bilateral brachial artery blood pressure was measured. In addition to standard echocardiography, the study included 3–5 cardiac cycles at frame rates of 50–70 frames per second on apical sections of four-, three- and two-chamber views that were stored digitally in original format for analysis. All strain analyses were performed using semiautomatic speckle tracking technology (EchoPAC203, GE Medical System, Milwaukee, Wisconsin) using the entire left ventricular model (the three apical views). Parts that were inadequately tracked were excluded. Three-dimensional full volume left ventricular acquisition using a maximum volume ratio matrix array sensor was attempted in all patients. The biplanar Simpson method was used to measure LVEF, while the measurement of GLS and myocardial work was based on automated function imaging (AFI). When the myocardial tracking was not accurate, the myocardium could be manually adjusted.

### Definitions of CTRCD

CTRCD (Zamorano et al., 2016) is defined as a reduction in LVEF of at least 5% from baseline to <53% (Negishi and Negishi, 2018) in absolute value, accompanied by symptoms or signs of heart failure, or a reduction in LVEF of at least 10% to an absolute value <53% without symptoms or signs of heart failure.

### Statistical Analysis

Statistical analysis involved the use of SPSS version 26.0 (SPSS, Chicago, IL) and GraphPad Prism 9.0 (GraphPad, United States). All measurement data were tested for normality and homogeneity of variance. Measurement data meeting the normal distribution were measured as the mean ± SD ( $\bar{X}$ ± s). Measurement data that did not meet the normal distribution were expressed as the median (interquartile range) [M (Q1∼Q3)], and the data between groups were compared using the Mann-Whitney U test. Paired t tests were used to compare the data from the same patient before and after chemotherapy. Survival analysis was performed by the Kaplan-Meier method, and the log-rank test was used for univariate analysis between groups. Multivariate survival analysis was performed by a Cox proportional risk regression model. *p* < 0.05 indicated that the difference was statistically significant.

## Results

### Patient Characteristics

A total of 206 breast cancer patients were screened from November 2018–January 2021. Among them, 79 were newly treated breast cancer patients who met the inclusion criteria and completed multicycle follow-up. All were female patients aged 22–66 years old, with a median age of 48 years old. There were 20 cases in the G1 group, 38 cases in the G2 group, and 21 cases in the G3 group. Among them, seven patients had hypertension, three patients had coronary heart disease, and three patients had diabetes. Eight patients were treated with dextroimide, and 26 patients were treated with other drugs (salvia miltiorrhiza polyphenol, cyclic adenosine glucamine, and safflower yellow). There were no significant differences in age, weight, height, BMI, body surface area (BSA), blood pressure, blood glucose or blood lipids among the different treatment groups (*p* > 0.05) (Table 1).

**TABLE 1:** Demographic and biochemical data in the different treatment groups [M (Q1∼Q3)].

	G1 (*n* = 20)	G2 (*n* = 38)	G3 (n = 21)	*p*
Age (years)	48(44–53.5)	49(43–56)	48(44–49)	0.711
Weight (kg)	61(54.25–67.38)	61.5(56.75–65)	62(56–75.5)	0.713
Height (cm)	160(158–163.75)	160(158–162)	160(157.5–165)	0.77
SBP (mmHg)	124.5(110.5–129.75)	122.5(116–138.25)	128(118–139.5)	0.603
DBP (mmHg)	75.5(65.75–79.75)	76(69–86)	74(67–82)	0.665
HR (bpm)	81.5(78–85)	81.5(72.5–90.5)	77(72.5–84.5)	0.678
BMI (kg/m^2^)	22.98(21.42–25.84)	23.98(21.93–26.51)	24.6(21.34–27.85)	0.665
BSA (m^2^)	1.67(1.56–1.74)	1.66(1.61–1.73)	1.69(1.59–1.84)	0.674
Glu (mmol/L)	4.94(4.65–5.38)	4.99(4.72–5.39)	5.11(4.64–5.56)	0.936
LDL-C (mmol/L)	2.46(2.06–2.77)	2.55(2.19–3.05)	2.22(2.02–3.13)	0.457
HDL-C (mmol/L)	1.33(1.19–1.54)	1.31(1.08–1.61)	1.3(1.05–1.63)	0.917
TG (mmol/L)	1.13(0.78–1.46)	1.05(0.84–1.65)	1.05(0.72–1.54)	0.773
TC (mmol/L)	4.27(3.92–4.56)	4.57(3.93–4.99)	4.29(3.95–4.83)	0.484

SBP, systolic blood pressure; DBP, diastolic blood pressure; HR, heart rate; BMI, body mass index; BSA, body surface area; Glu, blood glucose; TG, triglycerides; TC, total cholesterol. Data are expressed as [M (Q1∼Q3)].

### LVEF, GLS and GWI

**FIGURE 1:**
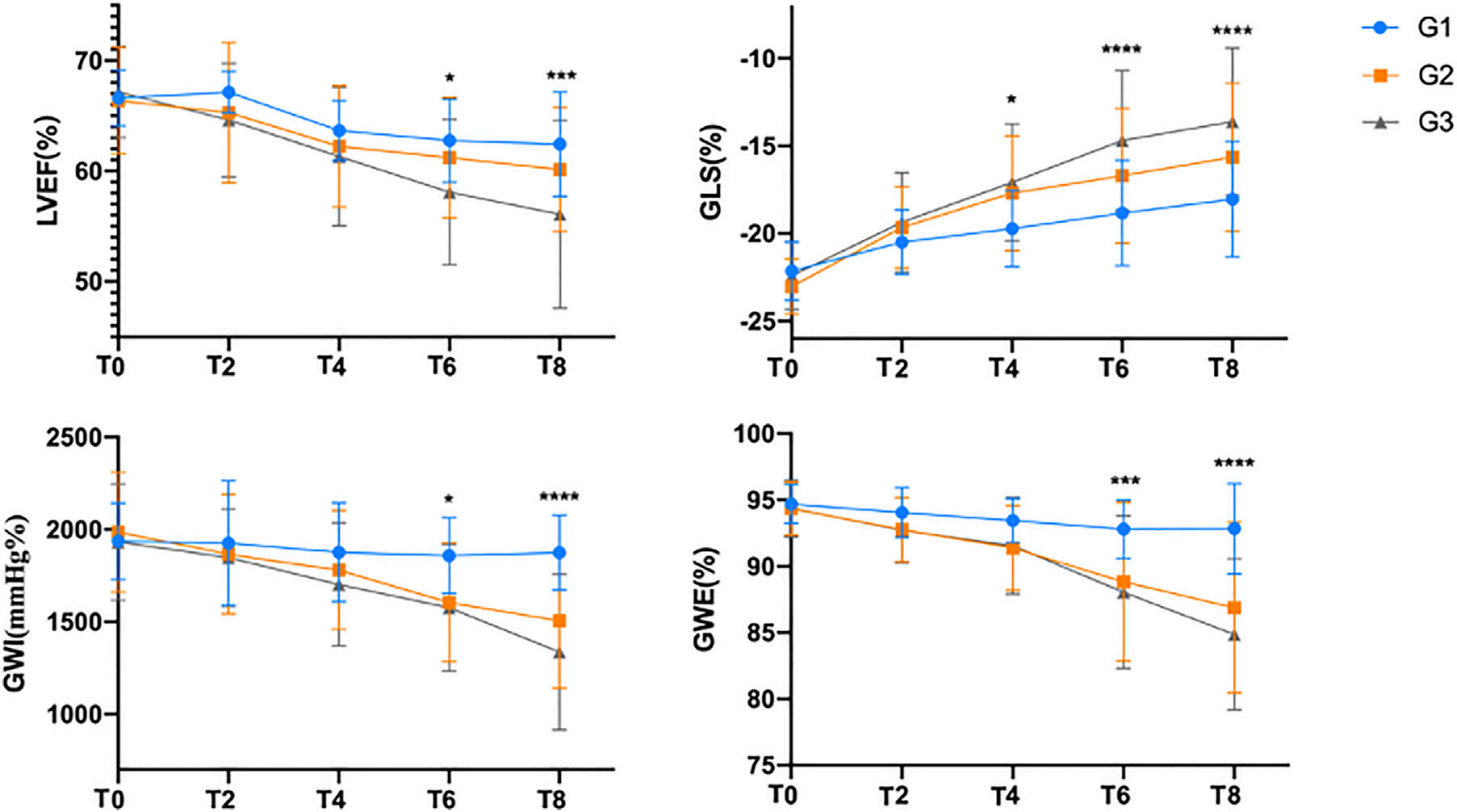
Comparison of the assessment of left ventricular function before and after chemotherapy.

Compared with the G1 group, GLS in T4 and LVEF, GWI and GWE in T6 were significantly decreased in the G2 and G3 groups (*p* < 0.05). With the progression of chemotherapy, the values of LVEF, GLS, GWI and GWE gradually decreased; GLS significantly decreased at T2; and LVEF, GWI and GWE significantly decreased at T4 (Figure 1).

**FIGURE 2:**
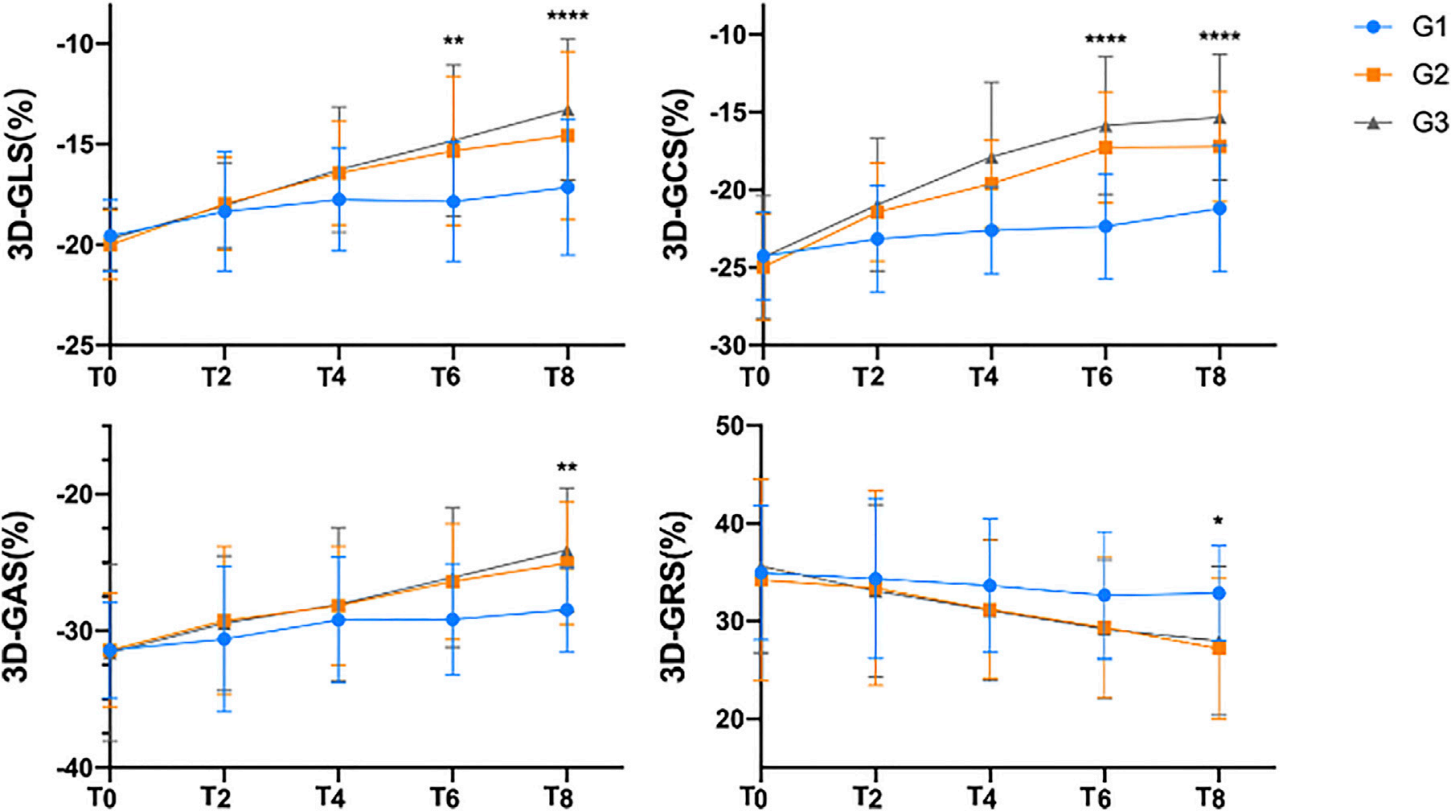
Comparison of the three-dimensional strain indices before and after chemotherapy.

The 3D-STE parameters of the research object are shown in Table 2. Compared with the basic condition before chemotherapy, 3D-GLS, 3D-GCS, 3D-GAS and 3D-GRS were significantly decreased (*p* < 0.05). The decrease in 3D-GLS and 3D-GCS in G2 and G3 was more obvious than that in G1, the changes were statistically significant in T6, and 3D-GAS and 3D-GRS were significantly changed in T8 (Figure 2).

**TABLE 2:** Left ventricular ultrasonography before and after chemotherapy ( $\bar{X}$ ± s).

	T0	T2	T4	T6	T8
LVEF (%)	66.65 ± 4.13	65.57 ± 5.25	62.34 ± 5.21#	60.73 ± 5.65#	59.43 ± 6.99#
GLS (%)	−22.6 ± 1.77	−19.79 ± 2.38#	−18.17 ± 3.04#	−16.96 ± 3.65#	−15.94 ± 4.03#
GWI (mmHg%)	1959.14 ± 294.34	1876.75 ± 309.1*	1783.51 ± 314.37#	1661.89 ± 321.25#	1552.58 ± 400.26#
GWE (%)	94.44 ± 1.91	93.08 ± 2.36#	91.95 ± 3.12#	89.62 ± 5.49#	87.84 ± 6.33#
3D-GLS (%)	−19.81 ± 1.69	−18.09 ± 2.41#	−16.72 ± 2.76#	−15.82 ± 3.71#	−14.86 ± 4.03#
3D-GCS (%)	−24.61 ± 3.42	−21.73 ± 3.62#	−19.87 ± 3.84#	−18.16 ± 4.51#	−17.7 ± 4.37#
3D-GAS (%)	−31.46 ± 4.73	−29.65 ± 5.23#	−28.39 ± 4.75#	−27 ± 4.58#	−25.65 ± 4.48#
3D-GRS (%)	34.8 ± 9.06	33.54 ± 9.12	31.8 ± 7.04*	30.14 ± 7.05#	28.85 ± 7.14#

LVEF, left ventricular ejection fraction; GLS, global longitudinal strain; GWI, global myocardial work index; GWE, myocardial work efficiency; 3D-GLS, left ventricular three-dimensional longitudinal peak strain; 3D-GCS, left ventricular three-dimensional peripheral peak strain; 3D-GAS, left ventricular three-dimensional area peak strain; 3D-GRS, left ventricular three-dimensional radial peak strain. *#p* < 0.05 compared with T0; *p < 0.01 compared with T0.

LVEF decreased in the course of chemotherapy, and the degree of decrease was G3 > G2 > G1. GLS decreased in the course of chemotherapy, and the degree of decrease was G3 > G2 > G1. GWI showed a downward trend in the course of chemotherapy, the change in GWI in the G1 group was not obvious, and the change range was G3 > G2. GWI showed a downward trend during chemotherapy, with no significant change in the G1 group, a similar decrease in the G2 group and G3 group at T4, and G3 > G2 after G6. Note: LVEF, left ventricular ejection fraction; GLS, left ventricular overall longitudinal strain; GWI, global myocardial work index; GWE, myocardial work efficiency; T0, baseline before chemotherapy; T2, after the second cycle of chemotherapy; T4, after the fourth cycle of chemotherapy; T6, after the sixth cycle of chemotherapy; T8, after the eighth cycle of chemotherapy. *p < 0.01 compared with G1.

3D-GLS, 3D-GCS, 3D-GAS and 3D-GRS all showed a downward trend during chemotherapy. The changes in 3D-GLS, 3D-GAS and 3D-GRS in the G3 group were significantly higher than those in the G1 and G2 groups (*p* < 0.05). The degree of decrease in 3D-GCS was G3 > G2 > G1. Note: 3D-GLS, left ventricular three-dimensional longitudinal peak strain; 3D-GCS, left ventricular three-dimensional peripheral peak strain; 3D-GAS, left ventricular three-dimensional area peak strain; 3D-GRS, left ventricular three-dimensional radial peak strain. *p < 0.01 compared with G1.

### CTRCD

During the follow-up, a total of nine patients developed CTRCD (see Supplementary Figure S1 for an example of image analysis). In the G1 group, one patient developed CTRCD at T8, and in the G2 group, one patient developed CTRCD at T2, one patient at T6, and one patient at T8. In the G3 group, one patient developed CTRCD at T2, two patients at T6, and two patients at T8.

The difference in clinical data between the event group (*n* = 9) and the nonevent group (*n* = 70) is shown in Table 3. There were no significant differences in age, systolic blood pressure, diastolic blood pressure, heart rate, blood glucose, HDL-C or TC between the two groups (*p* > 0.05), while BMI, BSA, LDL-C and TG in the event group were significantly increased (*p* < 0.05) (Table 3). L2v is defined as the rate of change of LVEF at T2, and G2v (GLS) compared with baseline, I2v (GWI), E2v (GWE), S2v (3D-GLS), C2v (3D-GCS), and A2v (3D-GAS) are similarly defined.

**TABLE 3:** Differences in clinical influencing factors at baseline between the CTRCD and non-CTRCD groups ( $\bar{X}$ ± s).

	CTRCD group (*n* = 9)	Non-CTRCD group (*n* = 70)	*p*
Age (years)	53.44 ± 9.4	48.09 ± 8.16	0.135
BMI (kg/m^2^)	29.46 ± 2.7	23.79 ± 3.19	<0.001
BSA (m^2^)	1.83 ± 0.14	1.66 ± 0.12	0.006
SBP (mmHg)	121.78 ± 10.5	127.7 ± 17.38	0.167
DBP (mmHg)	77.22 ± 8.26	76.01 ± 10.97	0.669
HR (bpm)	79.78 ± 7	80.99 ± 11.56	0.663
Glu (mmol/L)	5.08 ± 0.95	5.19 ± 0.86	0.742
LDL-C (mmol/L)	3.05 ± 0.49	2.47 ± 0.6	0.008
HDL-C (mmol/L)	1.37 ± 0.32	1.3 ± 0.94	0.353
TG (mmol/L)	1.3 ± 0.94	1.31 ± 0.76	0.971
TC (mmol/L)	5.08 ± 0.73	4.45 ± 0.8	0.034

SBP, systolic blood pressure; DBP, diastolic blood pressure; HR, heart rate; BMI, body mass index; BSA, body surface area; Glu, blood glucose; TG, triglycerides; TC, total cholesterol. Data are expressed as the mean ± SD.

Age, diastolic blood pressure, heart rate, E2v and A2v were not correlated with the occurrence of CTRCD. Systolic blood pressure, BMI, BSA, serum glucose levels, serum LDL-C levels, serum HDL-C levels, serum TC levels, serum TG levels, L2v, G2v, I2v, S2v and C2v were significantly correlated with the occurrence of end events (*p* < 0.05) (Supplementary Table S1).

**FIGURE 3:**
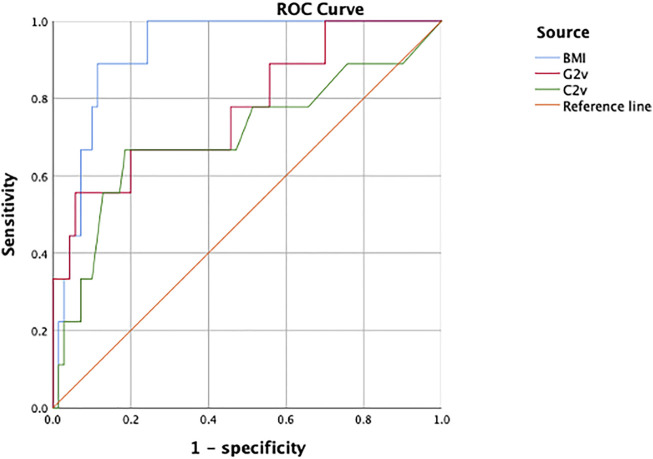
ROC curves were used to analyze the predictive value of BMI, G2v and C2v for cardiotoxicity.

The BMI level and G2v and C2v as parameters for predicting the occurrence of CTRCD in breast cancer patients after tumor treatment showed good predictive value, with AUCs of 0.922 (95% CI: 0.858–0.987), 0.776 (95% CI: 0.600–0.952) and 0.703 (95% CI: 0.488–0.918), respectively. At the 95th percentile risk threshold, the sensitivities were 88.9, 55.6, and 66.7%, and the specificities were 88.6, 94.3, and 81.4%, respectively. Among them, BMI is the optimal parameter. When BMI is higher than 27.03, the probability of CTRCD is 1.301 times that with a BMI less than 27.03. (Table 4, Figure 3).

**TABLE 4:** Analysis of the Cox regression results in the CTRCD and non-CTRCD groups.

	B	SE	Wals	*p*	HR (95.0% CI)
L2v (%)	0.123	0.116	1.116	0.291	1.131 (0.9–1.42)
G2v (%)	0.082	0.032	6.616	0.01*	1.086 (1.02–1.156)
I2v (%)	0.013	0.028	0.21	0.646	1.013 (0.958–1.071)
S2v (%)	3.847	2.085	3.403	0.065	46.854 (0.786–2791.451)
C2v (%)	5.512	2.299	5.75	0.016*	247.692 (2.737–22419.074)
A2v (%)	−0.053	2.969	0	0.986	0.949 (0.003–319.475)
BMI (kg/m^2^)	0.263	0.133	3.914	0.048*	1.301 (1.002–1.688)
BSA (m2)	0.849	2.775	0.094	0.76	2.336 (0.01–537.464)
LDL-C (mmol/L)	0.567	1.287	0.194	0.66	1.763 (0.142–21.945)
TC (mmol/L)	−0.054	0.905	0.004	0.953	0.948 (0.161–5.589)

SBP, systolic blood pressure; DBP, diastolic blood pressure; HR, heart rate; BMI, body mass index; BSA, body surface area; Glu, blood glucose; TG, triglycerides; TC, total cholesterol; L2v, rate of change of LVEF at T2; G2v, rate of change of GLS at T2; I2v, rate of change of GWI at T2; E2v, rate of change of GWE at T2; S2v, rate of change of 3D-GLS at T2; C2v, rate of change of 3D-GCS at T2; A2v, in the rate of change of 3D-GAS at T2. *p < 0.05.

The ROC curves of BMI, G2v and C2v for predicting the occurrence of cardiotoxicity are shown. The area under the curve of BMI (0.922) was better than that of G2v (0.776) and C2v (0.703), and its predictive value for the occurrence of cardiotoxicity was the highest.

## Discussion

There were several important findings in the present study. First, the GWI and GWE decreased with chemotherapy, but their sensitivity was lower than that of GLS. Second, 3D strain parameters such as 3D-GLS, 3D-GCS, 3D-GAS and 3D-GRS changed significantly in the course of chemotherapy but did not show obvious advantages over GLS. Third, BMI can be used as an independent factor to predict the occurrence of CTRCD.

In this cohort study, we analyzed the changes in GLS parameters and myocardial work parameters from echocardiography over the duration of tumor therapy and the role of these changes in predicting the occurrence of CTRCD. The results showed that GLS had statistically significant changes earlier than GWI. Theoretically, myocardial work is calculated based on the left ventricle pressure applied to the strain, under normal circumstances, the changes in myocardial work and ventricular strain will show better consistency. However, in this study, some patients developed hypertension after receiving chemotherapy treatment, which can balance the reduction of strain, so that the change in myocardial work index occurred after the reduction of strain. We plan to further explore these hypotheses in a follow-up study of hypertensive patients with tumors.

Chan et al. (2019) used LV-PSL to assess myocardial function in a group of coronary angiography patients. The study included control patients, patients with hypertension, and patients with cardiomyopathy. The results showed that the proportions of GCW and GWW increased in patients with hypertension and that GWE remained unchanged. In hypertensive patients with systolic blood pressure >160 mmHg, GLS and LVEF were normal and relatively unchanged, but the GWI was significantly higher than that of the control group, and the GWE of patients with cardiomyopathy decreased with the decrease in GLS and LVEF. The reduced efficiency reflects impairment in ventricular function. This study demonstrated that the GWI is more sensitive than GLS and LVEF in diseases associated with abnormal cardiomyocyte metabolism. However, the normal range of GWI and GWE in the Chinese population is still unknown due to the lack of large real-world studies evaluating cardiac function in normal controls. In this study, both GWI and GWE showed a downward trend with time after chemotherapy, and a significant change occurred after the second cycle of treatment, indicating that the use of chemotherapy drugs reduced the cardiac ejection work and the cardiac ejection work efficiency and that GWI and GWE, as corresponding measurement indicators, were sensitive to the response to chemotherapy drugs.

Evidence that 3D-STE can predict subsequent changes in 2D LVEF was first presented by Mornoş et al. (2014). In this study, 3D-GLS, 3D-GCS and 3D-GAS all decreased significantly after the second cycle of treatment, while 3D-GRS decreased after the fourth cycle, showing good consistency with the change in two-dimensional strain, and it could not be concluded that three-dimensional strain was superior to two-dimensional strain. Previous studies have found that three-dimensional ultrasound parameters (including 3D LVEF, GCS, longitudinal strain and principal strain) change more significantly than two-dimensional ultrasound parameters in breast cancer patients treated with anthracyclines, and these abnormalities persist after 2 years of follow-up (Kang and Scherrer-Crosbie, 2019). Some studies (Zhang et al., 2018) showed that the decline in the measured values of three-dimensional ultrasonic parameters was significantly correlated with the subsequent decline in LVEF, in which GLS and GCS were correlated with the changes in systolic function at the same time and thereafter. The follow-up time of this study was short, and the value of two-dimensional strain and three-dimensional strain in the evaluation of long-term prognosis needs further study.

A recent meta-study on the cardiotoxicity of anthracyclines (Nesser et al., 2009) showed that overweight and obesity were significantly associated with the risk of cardiotoxicity with anthracyclines and anti-Her-2 targeted therapy, consistent with the increased risk of higher body mass index in patients receiving anthracyclines and anthracyclines followed by trastuzumab in this study. Patients of normal weight had the lowest risk of cardiotoxicity, overweight patients had a moderate risk, and obese patients had the highest risk. Obesity was an independent risk factor for heart failure in a large epidemiological study conducted by Kenchaiah et al. (2002). Obesity can lead to excessive epicardial adipose tissue deposition and fibrosis, increase cardiac output and myocardial oxygen consumption, and increase the prevalence of heart failure (HF), especially HF with reduced ejection fraction (HFpEF) (Powell-Wiley et al., 2021), which may include left ventricular centripetal remodeling, right ventricular dilatation, and right ventricular three-compartment dysfunction. This process, combined with the toxic effect of chemotherapy drugs on the heart, makes obese patients more prone to cardiac dysfunction after chemotherapy.

Of the three groups, the results showed that the G3 group, which received anthracyclines in combination with targeted drugs, had the most significant changes in cardiac function. At present, anthracyclines are still essential in the treatment of breast cancer, but a small number of patients have been treated with targeted drugs alone. This study was limited by the patients’ economic burden and medical insurance status, and only a few patients were treated with double-targeted therapy alone (trastuzumab combined with pertuzumab). Therefore, patients who were treated with single-targeted therapy alone and double-targeted therapy alone were not compared and analyzed. We will conduct a comparative study of these two treatment regimens in the following study, aiming to explore whether pertuzumab can improve the treatment effect of patients while worsening their heart function.

## Conclusion

In tumor patients with little change in blood pressure, the predictive value of GLS on cardiac function is better than that of myocardial work parameters and 3D echocardiographic parameters. BMI, G2v and C2v were independent predictors of cardiac function impairment in breast cancer patients receiving chemotherapy, among which BMI was the best predictor. Further prospective studies in hypertensive patients are needed to evaluate the value of myocardial work parameters in predicting CTRCD.

**Table:** 

Index	Sensitivity (%)	Specificity (%)	AUC (95%CI)	Cutoff point	*P*
BMI (kg/m^2^)	88.9	88.6	0.922(0.858–0.987)	27.03	<0.001
G2v (%)	55.6	94.3	0.776(0.600–0.952)	22.7	<0.001
C2v (%)	66.7	81.4	0.703(0.488–0.918)	16.5	<0.001

## Data Availability

The original contributions presented in the study are included in the article/Supplementary Material, further inquiries can be directed to the corresponding authors.

## Abbreviations

CTRCD, heart dysfunction associated with cancer treatment; GLS, global longitudinal strain; LVEF, left ventricular ejection fraction; GWI, myocardial work index; GWE, myocardial work efficiency; BMI, body mass index; LV-PSL, left ventricular pressure-strain ring; T0, baseline before chemotherapy; T2, after the second cycle of chemotherapy; T4, after the fourth cycle of chemotherapy; T6, after the sixth cycle of chemotherapy; T8, after the eighth cycle of chemotherapy; SBP, systolic blood pressure; DBP, diastolic blood pressure; HR, heart rate; BSA, body surface area; Glu, blood glucose; TG, triglycerides; TC, total cholesterol; L2v, rate of change of LVEF at T2; G2v, rate of change of GLS at T2; I2v, rate of change of GWI at T2; E2v, rate of change of GWE at T2; S2v, rate of change of 3D-GLS at T2; C2v, rate of change of 3D-GCS at T2; A2v, rate of change of 3D-GAS at T2.
