# Supplementary material for: Assessment of Myocardial Work in Cancer Therapy-Related Cardiac Dysfunction and Analysis of CTRCD Prediction by Echocardiography
Source: Front Pharmacol. 2021 Nov 11;12:770580. doi: 10.3389/fphar.2021.770580 (PMC8632001; doi:10.3389/fphar.2021.770580)
Supplement: Supplementary file 1 [file Table1.DOCX]

**Supplementary Table 1**

**Supplementary Table 1 Correlation analysis of cardiovascular events associated with clinical factors**

| Index | X^2^ | p |
| --- | --- | --- |
| **Age (years)** | 33.583 | 0.298 |
| **BMI (kg/m^2^)** | 163.683 | ＜0.001* |
| **BSA (m^2^)** | 169.801 | ＜0.001* |
| **SBP (mmHg)** | 65.62 | 0.879 |
| **DBP (mmHg)** | 39.781 | 0.635 |
| **HR (bpm)** | 52.161 | 0.904 |
| **Glu (mmol/L)** | 120.967 | ＜0.001* |
| **LDL-C (mmol/L)** | 142.185 | 0.001* |
| **HDL-C (mmol/L)** | 81.599 | 0.005* |
| **TG (mmol/L)** | 141.991 | 0.005* |
| **TC (mmol/L)** | 172.478 | ＜0.001* |
| **L2v (%)** | 156.788 | ＜0.001* |
| **G2v (%)** | 156.788 | ＜0.001* |
| **I2v (%)** | 156.788 | ＜0.001* |
| **E2v (%)** | 35.539 | 0.155 |
| **S2v (%)** | 111.434 | ＜0.001* |
| **C2v (%)** | 85.532 | ＜0.001* |
| **A2v (%)** | 38.897 | 0.104 |

SBP, systolic blood pressure; DBP, diastolic blood pressure; HR, heart rate; BMI, body mass index; BSA, body surface area; Glu, blood glucose; TG, triglycerides; TC, total cholesterol; L2v, rate of change of LVEF at T2; G2v, rate of change of GLS at T2; I2v, rate of change of GWI at T2; E2v, rate of change of GWE at T2; S2v, rate of change of 3D-GLS at T2; C2v, rate of change of 3D-GCS at T2; A2v, rate of change of 3D-GAS at T2.
