# Supplementary figures and images for: Assessment of Myocardial Work in Cancer Therapy-Related Cardiac Dysfunction and Analysis of CTRCD Prediction by Echocardiography
Source: Front Pharmacol. 2021 Nov 11;12:770580. doi: 10.3389/fphar.2021.770580 (PMC8632001; doi:10.3389/fphar.2021.770580)

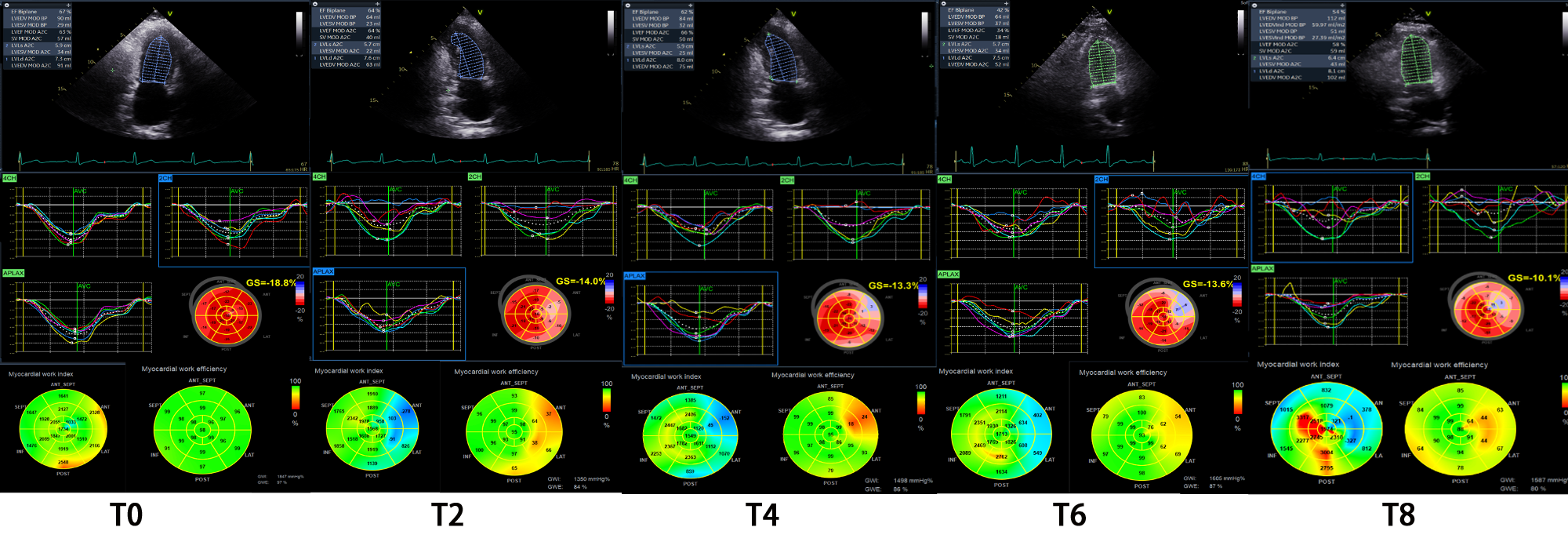

Supplement: Supplementary file 2 [file Image1.TIF]
